# Chronic myeloid leukaemia with marked thrombocytosis

**DOI:** 10.1002/jha2.135

**Published:** 2020-11-13

**Authors:** Jun Ooi, Tomonori Sato

**Affiliations:** ^1^ Department of Hematology/Oncology Teikyo University School of Medicine Tokyo Japan; ^2^ Saiseikai Kawaguchi General Hospital Saitama Japan

A 47‐year‐old male presented with bruising on the right thigh. His full blood count (FBC) showed leucocyte, 12.80 × 10^9^/L; haemoglobin 142 g/L; platelets, 5199 × 10^9^/L. A peripheral blood film revealed marked thrombocytosis with micromegakaryocytes and giant platelets (Figure 1, upper: May‐Giemsa stain, 40× objective). He had no splenomegaly. Bone marrow aspirate revealed hypercellularity with marked proliferation of variable sized megakaryocytes (Figure 1, lower left: May‐Giemsa stain, 40× objective). Myeloid/erythroid ratio was 2.5. He was tentatively diagnosed with essential thrombocythemia and received hydroxycarbamide, however, bone marrow cytogenetics showed t(9;22) (Figure 1, lower right) and molecular analysis revealed a BCR‐ABL1 transcript. The diagnosis of chronic myeloid leukaemia (CML) was confirmed. He received dasatinib and his FBC promptly normalized. He achieved a major molecular response in 3 months with dasatinib treatment and has maintained a deep molecular response for 3 years. Our case showed the importance of chromosome and molecular analysis in the diagnosis of myeloproliferative neoplasms. To date, only one paediatric CML case with marked thrombocytosis more than 5000 × 10^9^/L has been reported.

**FIGURE 1:**
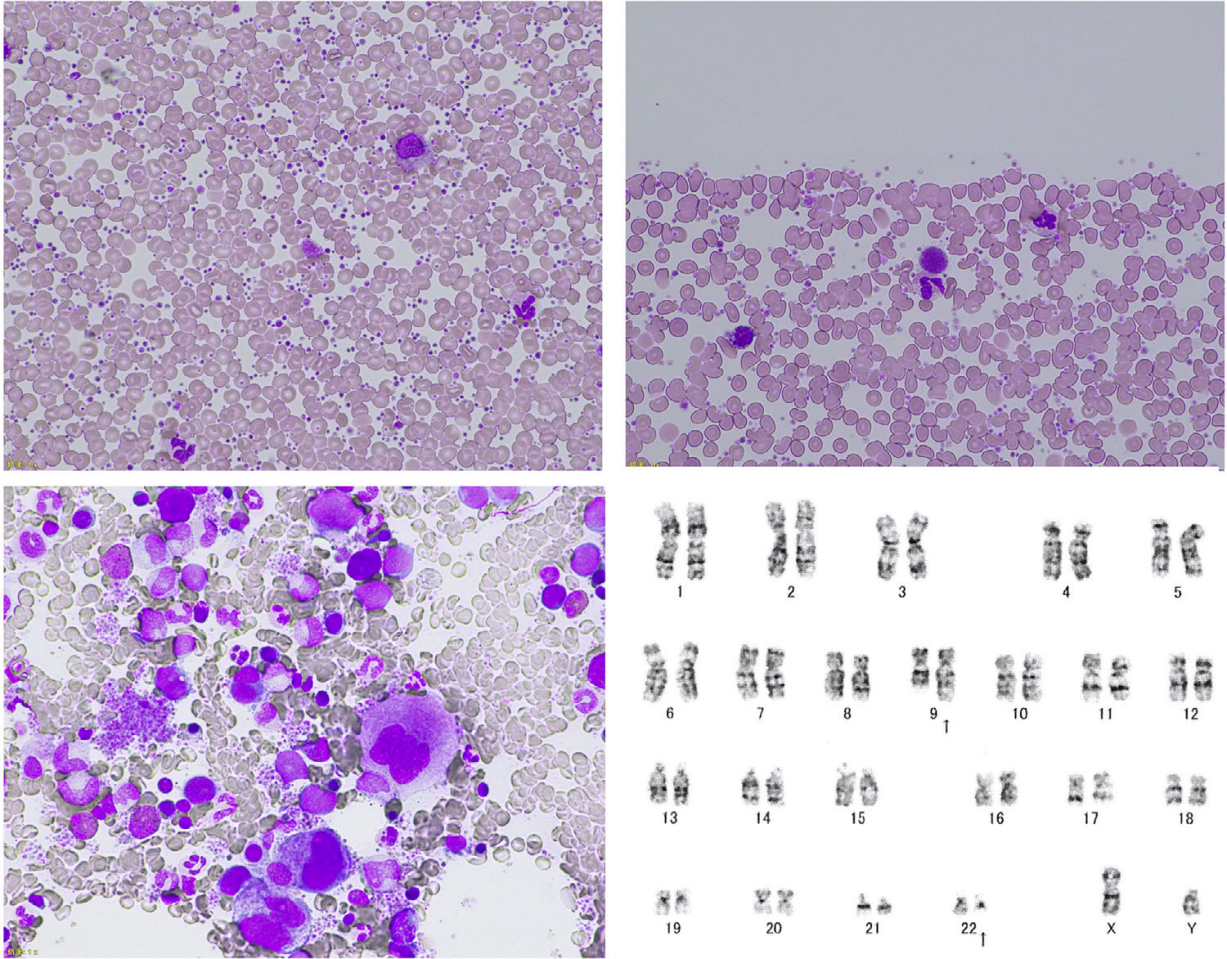
A peripheral blood film revealed marked thrombocytosis with micromegakaryocytes and giant platelets (upper). Bone marrow aspirate revealed hypercellularity with marked proliferation of variable sized megakaryocytes (lower left). Bone marrow cytogenetics showed t(9;22) (lower right).

## AUTHOR CONTRIBUTIONS

Jun Ooi wrote the paper, and Jun Ooi and Tomonori Sato performed the research.

## CONFLICT OF INTEREST

The authors declare that they have no conflict of interest.

## INFORMED CONSENT

Informed consent was obtained from our patient included in this article.

